# Inflammation as a contributing factor among postmenopausal Saudi women with osteoporosis

**DOI:** 10.1097/MD.0000000000005780

**Published:** 2017-01-27

**Authors:** Nasser M. Al-Daghri, Ibrahim Aziz, Sobhy Yakout, Naji J. Aljohani, Yousef Al-Saleh, Osama E. Amer, Eman Sheshah, Ghaida Zakaria Younis, Fahad Badr M. Al-Badr

**Affiliations:** a Prince Mutaib Chair for Biomarkers of Osteoporosis, Biochemistry Department, College of Science, King Saud University; b Obesity, Endocrine and Metabolism Center, King Fahad Medical City, Faculty of Medicine; c College of Medicine, King Saud bin Abdulaziz University for Health Sciences; d Diabetes Care Center, King Salman Bin Abdulaziz Hospital; e Department of Radiology and Medical Imaging, King Khalid University Hospital, King Saud University, Riyadh, Saudi Arabia.

**Keywords:** bone turnover marker, 25-OH vitamin D, osteoporosis, postmenopausal, proinflammatory cytokine

## Abstract

Postmenopausal osteoporosis is an important metabolic bone disease characterized by rapid bone loss occurring in the postmenopausal period. Recently, the most prevalent form of clinically significant osteopenia and osteoporosis involves various inflammatory conditions. The aim of the study is to evaluate the association between proinflammatory markers (interleukin [IL]-1β, IL-6, TNF-α) with bone turnover markers (BTMs) in postmenopausal Saudi women with and without osteoporosis. A total of 200 postmenopausal Saudi women ≥50 years old, 100 with osteoporosis and 100 without osteoporosis (control) were recruited under the supervision of qualified physicians in King Salman Hospital and King Fahd Medical City, Riyadh, Saudi Arabia. Serum tumor necrosis factor alpha (TNF-α), IL-1, IL-4, IL-6, and parathyroid hormone (PTH) were determined using Luminex xMAP technology. N-telopeptides of collagen type I (NTx) was assessed using ELISA, 25(OH) vitamin D and osteocalcin were determined using electrochemiluminescence, serum calcium and inorganic phosphate (Pi) were measured by a chemical analyzer. Serum IL-1β, IL-6, NTx, and PTH levels in women with osteoporosis were significantly higher than controls. Although IL-4 and osteocalcin were significantly lower than controls. IL-1β and TNF-α were positively associated with NTx in osteoporosis women. TNF-α, IL-6, and TNF-α were positively correlated with IL-lβ in both groups. A significant negative correlation between osteocalcin and IL-1β in healthy women and women with osteoporosis were observed. Findings of the present study implicate a role for cytokine pattern-mediated inflammation in patients with osteoporosis.

## 1 Introduction

Osteoporosis is a progressive bone disease and a global problem estimated to affect 200 million people worldwide.^[1]^ In the year 2000 alone there were an estimated 9 million new osteoporotic fracture cases. The Americas and Europe accounted for 51% of all these fractures, the remainder occurred in Southeast Asia and the Western Pacific region.^[2]^

Postmenopausal women have a higher risk for osteoporosis due to estrogen hormone decline which contributes to the development of osteoporosis through spontaneous increases in proinflammatory and proosteoclastic cytokines such as interleukin (IL)-6, tumor necrosis factor alpha (TNF-α), and IL-1 that lead to enhance the ability of osteoclasts to absorb bone.^[3–5]^

In Saudi Arabia, the prevalence of osteoporosis (≥50 years) is as high as 44.5% in Saudi women and 33.2% in Saudi men.^[6]^ Moreover, the incidence of fragility fractures jumped from 2.9/1000 in 1999 to 6/1000 in 2007 at an annual cost of SR4.27 billion.^[7,8]^ According to El-Desouki,^[9]^ the prevalence of postmenopausal osteoporosis in the age between 50 and 79 years was 57.5%. Another study by Oommen and AlZahrani^[10]^ showed that 58% of Saudi women had low bone mineral density (BMD) (18% had osteoporosis and 40% had osteopenia).

Epidemiological studies have identified higher incidence of osteoporosis in various inflammatory conditions such as ankylosing spondylitis, rheumatoid arthritis, and systemic lupus erythematosus.^[11–13]^ This association was also observed clinically where the degree of osteoporosis was equivalent to the extent of inflammation. The occurrence of inflammation is indicated by the presence of inflammatory markers such as cytokines and C-reactive protein. Biochemical studies have demonstrated elevation of proinflammatory cytokines TNF-α and IL-6 in arthritic disease such as gouty arthritis, rheumatoid arthritis, and psoriatic arthritis.^[14,15]^

An obvious relationship between inflammation and osteoporosis was seen in rheumatoid arthritis, whereby proinflammatory cytokines were released causing bone loss around the affected joints and increase of bone turnover markers (BTMs).^[16,17]^ Proinflammatory cytokines may contribute to bone loss by osteoclasts which activated by receptor activator of nuclear factor κB ligand (RANKL) leading to osteoporosis.^[18]^

During bone resorption, osteoclasts produce N-telopeptides of type I collagen (NTx) and carboxy-terminal telopeptides of collagen type I into the circulation.^[19]^ However, osteoblasts synthesize a number of molecules that reflect bone formation rates such as osteocalcin, bone specific alkaline phosphatase, and procollagen 1 carboxyterminal propeptide.^[19]^

Bone markers are not used for the diagnosis of osteoporosis because there is a great overlap between levels of osteoporotic and nonosteoporotic patients.^[20]^ However, they can be helpful in estimating bone turnover rates. The study aims to evaluate the associations between proinflammatory markers (IL-1β, IL-6, and TNF-α) with BTMs in postmenopausal Saudi women with and without osteoporosis.

## 2 Methods

### 2.1 Subjects

This study included a total of 200 Saudi postmenopausal women aged ≥50 years old (N = 100 with osteoporosis and N = 100 without osteoporosis) recruited from King Salman Hospital and King Fahd Medical City, Riyadh, Saudi Arabia. All measurements were performed systematically between August, 2013 and September, 2014. The subject's history was taken from a generalized questionnaire including age, age of menarche, age of menopause, family history for osteoporosis, medical history, disease status, etc. Ethics approval was granted by The Ethics Committee of The College of Science, King Saud University, Riyadh, Kingdom of Saudi Arabia, No 8/25/36516.

BMD (g/cm^2^) was measured for femoral neck by dual-energy X-ray absorptiometry DXA (Hologic QDR 2000 Inc., Woltham, MA). The diagnostic criteria of osteoporosis based on the T-score for BMD established according to The World Health Organization definitions that use T score assessment, T-score value of −2.5 SD or below the mean for a young healthy adult woman indicate osteoporosis. T-score value between −1.0 and −2.5 SD indicates osteopenia and T- score value of −1.0 SD or more as normal. Patients with acute medical conditions that require immediate medical attention and with other associated diseases and inflammatory condition (illnesses fall beyond routine cold or flu infections) and no history of any other bone disease were excluded.

### 2.2 Anthropometry and blood collection

Subjects’ anthropometry included height and weight were determined using standardized conventional methods in light clothes and without shoes, waist and hip circumference were obtained using a standardized nonstretchable fiber measuring tape, waist-to-hip ratio (WHR) was calculated as the ratio of waist and hip circumferences, mean blood pressure (systolic and diastolic in mm Hg) were measured. Body mass index (BMI) was calculated as body weight divided by height squared (kg/m^2^).

### 2.3 Sample analysis

Five (5) mL of fasting venous blood samples were collected in tubes without anticoagulant (serum separator tubes) were collected in the morning on an assigned day. Samples were then left to clot at room temperature for 30 minutes, and then were centrifuged at 5000 round per minutes (RPM) for 10 minutes. After that the collected serums were transferred to prelabeled plain tubes, stored in ice, and then transported to The Biomarkers Research Program in King Saud University, Riyadh, Kingdom of Saudi Arabia, for immediate storage at −80 °C until analysis.

Serum cytokine concentration (IL-1β, IL-6, and TNF-α) as well as PTH was quantified using Luminex Multiplex Assay System (Luminex Inc) kits purchased from EMD Millipore's Milliplex MAP. IL-1β (intra- and interassay coefficient of variation [CV]) was 7% and 9%, respectively. IL-6 (intra- and interassay CV were 7% and 13%, respectively). TNF-α (intra- and interassay CV were 8% and 7%, respectively). PTH (intra- and interassay CV were 4% and 9%, respectively).

Serum NTx was measured using a competitive-inhibition enzyme-linked immunosorbent assay (ELISA) kits purchased from Alere Scarborough, Inc. (10 Southgate Road Scarborough, ME). Intra- and interassay CV were 4.6% and 6.9%, respectively. Osteocalcin and 25-OH vitamin D were determined by Electrochemiluminescence Immunoassay, kit purchased from (Roche Diagnostics, Mannheim, Germany). Osteocalin, intra- and interassay CV were 4% and 6.5%, respectively. Biomarkers Research Program is an accredited laboratory by the 25-OH vitamin D External Quality Assessment Scheme (DEQAS). Serum calcium and Pi were carried out using a chemical analyzer (Konelab, Espoo, Finland) kit purchased from Thermo Fisher Scientific Oy, ref 981367. Calcium, intra- and interassay CV were 0.2% and 0.1%, respectively. Pi, intra- and interassay CV was 1.9% and 4.7%, respectively.

### 2.4 Statistical analysis

Data were entered and analyzed using SPSS version 16.5 (SPSS Inc., Chicago, IL). Continuous data were represented by mean ± SD for variables following Gaussian distribution and non-Gaussian variables. Categorical data were represented by frequencies and percentages. Each continuous variable was checked for normality by Kolmogorov–Smirnov test. Differences between groups (cases and control) were done using Student *t* test. For non-Gaussian variables and Mann–Whitney *U* test were determined to compare groups. A *P* value <0.05 was considered as statistically significant.

## 3 Results

The general characteristics of subjects are summarized in (Table 1). The 100 Saudi postmenopausal women without osteoporosis were 55.21 ± 8.77 years old, and 100 with osteoporosis were 56.22 ± 7.16 years old participated in the study. There was a significant difference s between patients with osteoporosis and controls with respect to menarche age, weight, waist, hip circumference, and BMI (*P* = 0.031), (*P* = 0.000), (*P* = 0.038), (*P* = 0.000), and (*P* = 0.000), respectively. The BMD volume of both dual femur as well as lumbar spine were significantly lower in patients with osteoporosis than controls (*P* = 0.000).

**Table 1:**
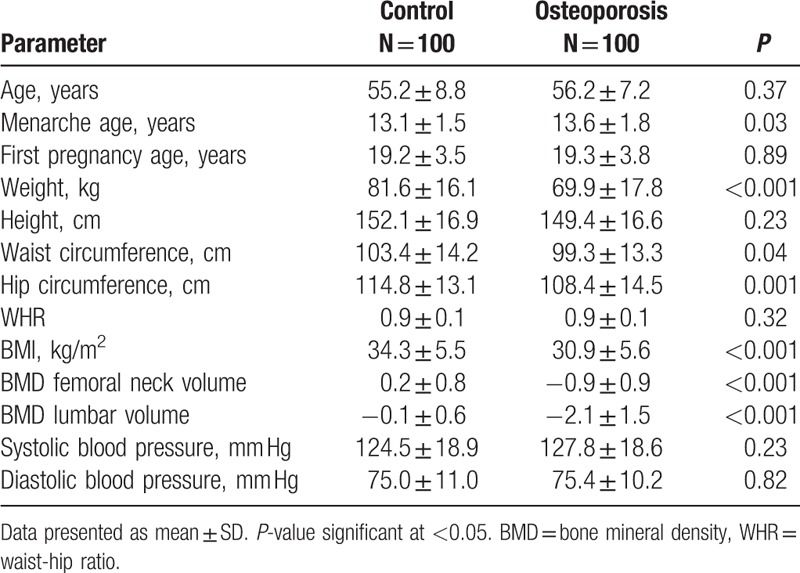
General characteristics of subjects.

The biochemical metabolic markers of the case–control study subjects are summarized in (Table 2). IL-lβ, IL-6, PTH, and NTx serum levels were significantly elevated in patients more than controls (*P* = 0.025), (*P* = 0.044), (*P* = 0.038), and (*P* = 0.034), respectively. However, levels of IL-4 and osteocalcin were significantly lower in women with osteoporosis (*P* = 0.007) and (*P* = 0.028), respectively.

**Table 2:**
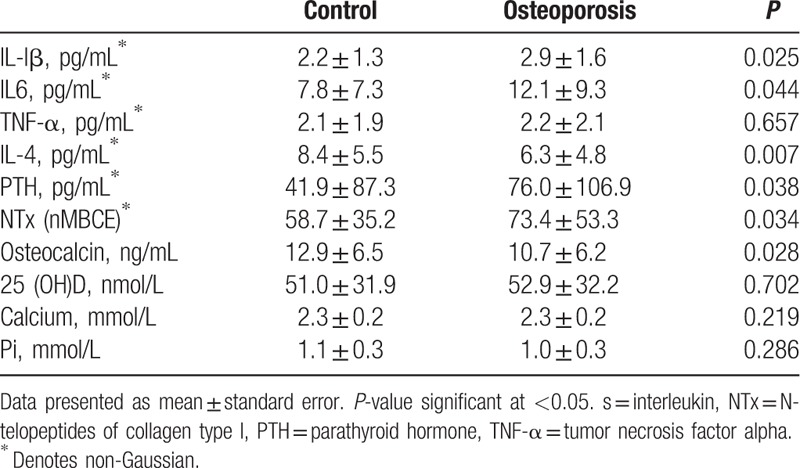
Biochemical metabolic markers of subjects.

The associations of proinflammatory and biochemical metabolic markers of subjects are summarized in (Table 3). In the univariate correlation analysis, IL-lβ was positively correlated with IL-6, TNF-α, and PTH (*r* = 0.584, *P* **<** 0.001), (*r* = 0.761, *P* **<** 0.001), and (*r* = 0.807, *P* **<** 0.001), respectively, and negatively with IL-4 and osteocalcin (*r* = −0.387, *P* **<** 0.001), and (*r* = −0.405, *P* **<** 0.001), respectively. There was also significant positive association between IL-6 and TNF-α as well as PTH (*r* = 0.347, *P* **<** 0.001) and (*r* = 0.313, *P* **<** 0.001), respectively, and negative association with IL-4 (*r* = −0.396, *P* **<** 0.001), respectively. TNF-α also showed statistically significant positive relationship with PTH (*r* = 0.680, *P* **<** 0.001), and negative association with osteocalcin (*r* = −0.263, *P* **<** 0.001). However, IL-4 was significant negative association with PTH as well as NTx (*r* = −0.277, *P* **<** 0.001) and (*R* = −0.314, *P* **<** 0.001), respectively. PTH showed a positively associated with respect osteocalcin (*r* = 0.320, *P* **<** 0.001).

**Table 3:**
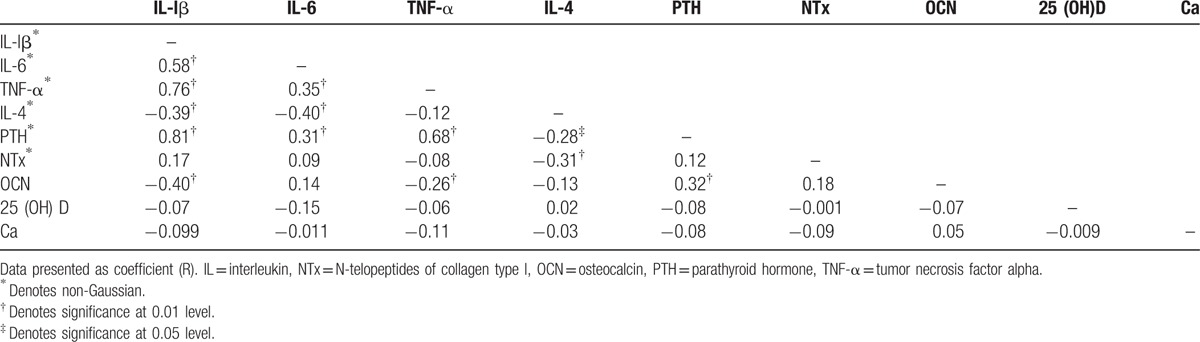
Associations of proinflammatory cytokines and biochemical metabolic markers of study subjects.

The associations of proinflammatory and biochemical metabolic markers of controls are summarized in (Table 4). Serum IL-lβ was positively associated with IL-6, TNF-α, and PTH (*r* = 0.578, *P* **<** 0.001), (*r* = 0.802, *P* **<** 0.001), and (*r* = 0.781, *P* **<** 0.001), respectively, and negatively associated with IL-4 and osteocalcin (*r* = −0.402, *P* **<** 0.05) and (*r* = −0.554, *P* **<** 0.001), respectively. There was also significant positive correlation between IL-6 with TNF-α and PTH (*r* = 0.419, *P* **<** 0.001) and (*r* = 0.369, *P* **<** 0.001), respectively, and negative correlation with IL-4 (*r* = −0.425, *P* **<** 0.05). TNF-α also positively correlated with PTH (*r* = 0.719, *P* **<** 0.001), there was also positively correlated with osteocalcin (*r* = −0.446, *P* **<** 0.001). However, a significant negative correlation was found between IL-4 with PTH, as well as NTx (*r* = −0.310, *P* **<** 0.05) and (*r* = −0.382, *P* **<** 0.001), respectively. Although PTH showed positive correlation with osteocalcin (*r* = 0.350, *P* **<** 0.001).

**Table 4:**
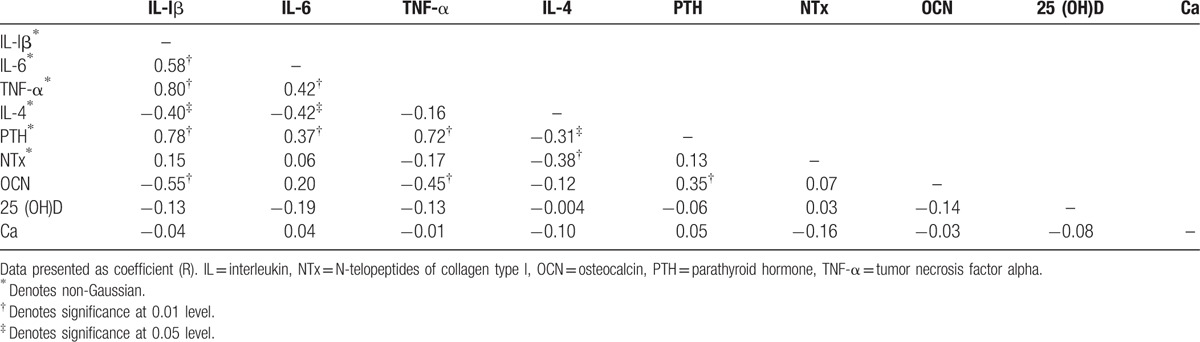
Associations of cytokine profile and biochemical metabolic markers of control group.

The associations of proinflammatory and biochemical metabolic markers of women with osteoporosis group are summarized in (Table 5). In the univariate correlation analysis, serum levels of IL-lβ showed a significant positive correlation with NTx (*r* = 0.301, *P* **<** 0.05). IL-lβ was also a significant positive correlations were showed with IL-6, TNF-α, and PTH (*r* = 0.615, *P* **<** 0.001), (*r* = 0.692, *P* **<** 0.001), and (*r* = 0.844, *P* **<** 0.001), respectively, and negatively correlated with osteocalcin (*r* = −0.446, *P* **<** 0.001). IL-6 showed negatively correlated with IL-4 (*r* = −0.348, *P* **<** 0.05) variable. Serum levels of TNF-α showed a significant positive correlation with NTx (*r* = 0.660, *P* **<** 0.001). TNF-α was also positively correlated with PTH and calcium (*r* = 0.627, *P* **<** 0.001) and (*r* = 0.276, *P* **<** 0.05), respectively. IL-4 showed significant positive association with osteocalcin (*r* = 0.138, *P* **<** 0.05). However, PTH was positively associated with calcium (*r* = 0.270, *P* **<** 0.05). NTx was positively associated with osteocalcin (*r* = 0.321, *P* **<** 0.05).

**Table 5:**
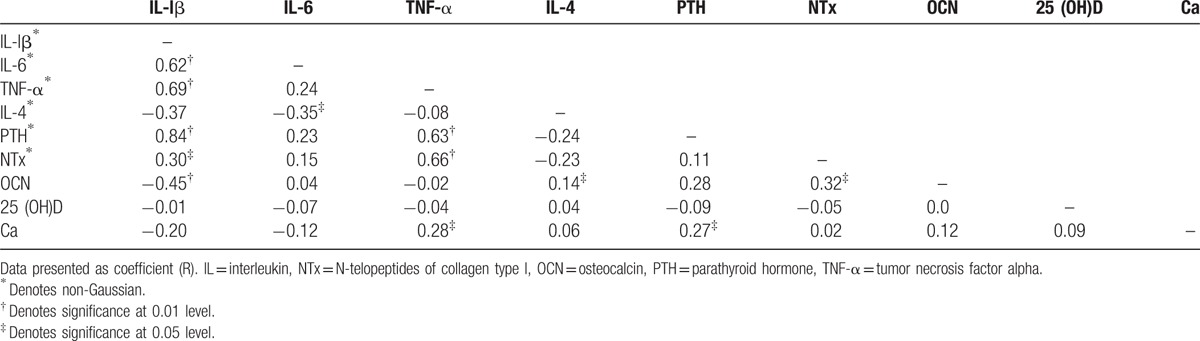
Associations of cytokines profile, and biochemical metabolic markers of cases cohort.

## 4 Discussion

The purpose of this study was to investigate the association between proinflammatory cytokines with BTMs in postmenopausal Saudi women with and without osteoporosis. Significantly lower BMI in postmenopausal women with osteoporosis than controls were observed, which is in agreement with the previous observations of Reid.^[21]^ Other studies suggested no significant difference between BMI and osteoporosis.^[22]^ On the other hand, there is some evidence that various cytokines as TNF-α and IL-6 are increased in obesity and decreased with weight loss, and it is believed that they play a role in causing insulin resistance. The main source of proinflammatory cytokines in obesity is the adipose tissue; they are mainly produced by infiltrating macrophages.^[23,24]^

The BMD lumbar volume was also significantly lower in postmenopausal women with osteoporosis than controls. There was no significant difference in systolic and diastolic blood pressure between patients and controls, consistent with Mussolino and Gillum^[25]^ reported that there were no significant associations between BMD and hypertension in men or women of African American or White ethnicity by using the National Health and Nutrition Examination Survey data.

Proinflammatory cytokines (IL-1β and IL-6) were significantly elevated in patients than controls, consistent with other studies that investigated the relationship between osteoporosis and inflammation.^[26,27]^ Proinflammatory cytokines are frequently regulated in cascades, where induction of the early cytokines serves to stimulate the release of later cytokines. The specificity for cytokines response is provided by unique cytokine receptors. Cells that express a functional receptor for a cytokine can respond to the presence of that cytokine. IL-1 stimulates the release of IL-2, IL-6, and TNF-α.^[28,29]^ A major consequence of estrogen deficiency in postmenopausal women and loss of ovarian function would be expected to promote signaling and gene expression cascade of major proinflammatory cytokines that directly induce early osteoclast precursor formation, that is, macrophage colony-stimulating factor (M-CSF) and IL-6. Spontaneous increases in these cytokines may be further enhanced by the parallel increases in TNF-α and IL-1 with menopause, which are potent enhancer of M-CSF and IL-6.^[30,31]^

Evidence from animal and in vitro studies suggests that increases in these cytokines promote bone resorption through several mechanisms, including increasing osteoclast differentiation, activation, and survival; enhancing RANKL expression; and inhibiting osteoblast survival.^[32–34]^ TNF-α has long been implicated in osteoclast formation in postmenopausal osteoporosis through 2 mechanisms, the 1st process occurs when stromal cells are exposed to TNF-α and to amplify RANKL, M-CSF, and IL-1, which enhance osteoclast activation and differentiation. The 2nd mechanism suggested that TNF-α may promote osteoclast formation by directly stimulating its precursors in the absence of stromal cells responsive to the cytokine, perhaps through activation of transforming growth factor-β.^[35]^ IL-1β has also been shown that osteoclast is one of the target cells for IL-1β, it provides an important stimulus the formation and activity of osteoclasts, leading to excessive bone resorption. Furthermore, Lee et al^[36]^ reported that the presence of osteoblast and stromal cells was crucial in the formation of osteoclasts by IL-1β. Moreover, Xu et al^[37]^ found that rat osteoclasts expressed mRNA to IL-1β receptors.^[37]^ IL-1β may also act in the formation of osteoclasts by nuclear factor kappa-light-chain-enhancer of activated B cells and prevents its apoptosis.^[38]^ IL-6 is another cytokine that produced by many different cell types as monocytes and stromal cells, when they exposed to various types of stimulation, like endotoxin, IL-1, and TNF-α.^[39]^ IL-6 was also reported to be produced by osteoblasts when stimulated by PTH, 25(OH) vitamin D3, and growth factor.^[40]^ In addition, IL-6 can promote the differentiation of osteoclasts from its precursor by stimulating RANKL expression, IL-6 also directly enhance osteoclast activity by RANKL-independent mechanisms.^[41]^ Moreover, IL-6 may directly extend the lifespan of the osteoclasts by inhibiting osteoclast apoptosis.^[42]^

Mature osteoclasts are responsible for the dissolution and of resorbing bone by attaching to the surface and then secreting protons into an extracellular compartment formed between osteoclast and bone surface. This secretion is necessary for bone mineral solubilization and the digestion of organic bone matrix by acid proteases.^[43]^

There is a significant positive correlation between IL-1β, TNF-α, and NTx in women with osteoporosis similar to previous studies.^[44,45]^ TNF-α and IL-1β are known to inhibit bone formation by depressing osteoblast activity, synthesis of collagen, and secretion and mineralization of the extracellular bone matrix.^[46,47]^ These results are also consistent with a significant role of proinflammatory cytokines in the inflammatory state present in osteoporosis.

Osteocalcin and NTx levels as BTMs were evaluated in both groups. The level of NTx was significantly higher in the osteoporosis group as compared to controls, and the level of osteocalcin was significantly lower than controls. These results are in agreement with studies done on osteocalcin and NTx.^[48]^ The exact mechanism of osteocalcin which is produced by osteoblasts during the bone formation process in bone is still unclear.^[19]^ Bone degradation process involves breakdown of type I collagen as carboxy-terminal telopeptides of collagen type I and NTx, which are used to determine the rate of bone resorption.^[20,49]^ The previous reports are in agreement with our finding since NTx was higher in the women with osteoporosis than healthy women. These results support a high rate of bone resorption in women with osteoporosis. Moreover, these results are also consistent with previous study suggested that low BMD in the presence of high BTMs is more predictive of fracture than either risk factor alone.^[50]^

A significantly higher PTH in the women with osteoporosis as compared to controls was observed. PTH indirectly stimulates bone resorption by acting upon its receptors on osteoblasts. PTH acts on PTH1R on osteoclasts. Activation of PTH1R initiates several parallel signaling pathways, which include Ca^2+^/PKC pathway and G subunit (Gs) protein Gs protein to increase protein kinase A activity and thereby cAMP-mediated transcriptional activity by insulin-like growth factor-1.^[51,52]^ Together protein kinase A, insulin-like growth factor-1, and PKC result in an increase in RANKL and CSF, leading to stimulate of osteoclastogenesis.^[51,52]^

Moreover, a significant positive correlation was found between PTH and IL-lβ, IL-6 as well as TNF-α in patients and controls, consistent with previous studies.^[27]^ In addition, it has been demonstrated that cytokines as IL-6, which potently induces osteoclastogenesis, is produced by osteoblastic cells in response to PTH.^[53]^ On the other hand, there is some evidence that various cytokines induce the expression of PTH-related protein/peptide. Proinflammatory cytokines induced expression of PTH-related protein/peptide in spleen stromal cells and hepatocytes during endotoxemia.^[54,55]^

This study acknowledges a few limitations. Small sample size and cross-sectional based study cannot suggest any causal and temporal correlations. Large-scale prospective studies are required to determine the exact predictive value of these biomarkers. Subjects were limited to women as contributing factors to osteoporosis maybe different in men due to sexual dimorphic expression of inflammation.^[56]^

## 5 Conclusion

In summary, findings of the present study implicate a role for cytokine-mediated inflammation in patients with osteoporosis. Further studies are needed to improve our understanding of cytokine pattern implication in inflammation in large scale to estimate the association of cytokine with BTMs and bone metabolism.

## Acknowledgments

The authors thank National Plan for Science, Technology and Innovation (MAARIFAH), King Abdulaziz City for Science and Technology, Kingdom of Saudi Arabia, Award number (11-MED-2113-02) for the support.
